# A Simplified Method for Calculating Surface Area of Mammalian Erythrocytes

**DOI:** 10.3390/mps7010011

**Published:** 2024-01-25

**Authors:** Ion Udroiu

**Affiliations:** Dipartimento di Scienze, Università degli Studi “Roma Tre”, 00146 Rome, Italy; ion.udroiu@uniroma3.it

**Keywords:** MCV, RBC, red blood indices, volume, area, mammals

## Abstract

Knowledge of the geometric quantities of the erythrocyte is useful in several physiological studies, both for zoologists and veterinarians. While the diameter and volume (MCV) are easily obtained from observations of blood smears and complete blood count, respectively, the thickness and surface area are instead much more difficult to measure. The precise description of the erythrocyte geometry is given by the equation of the oval of Cassini, but the formulas deriving from it are very complex, comprising elliptic integrals. In this article, three solids are proposed as models approximating the erythrocyte: sphere, cylinder and a spheroid with concave caps. The volumes and surface areas obtained with these models are compared to those effectively measured. The spheroid with concave caps gives the best approximation and can be used as a simple model to determine the erythrocyte surface area. With this model, a simple method that allows one to estimate the surface area by knowing only the diameter and MCV is proposed.

## 1. Introduction

Hematological parameters have been widely examined in several kinds of studies on mammalian species. Blood indices have been used to assess the health status of wild populations [1,2,3,4,5], but also to investigate allometric [6,7,8,9] and phylogenetic relationships [10,11]. Hematological studies have also been instrumental in studying behavioral and ecological characteristics, such as burrowing [12], diving [13], flight [14] and adaptation to high altitude [15,16].

Erythrocytes, or red blood cells (RBCs), make up more than 80% of all cells in the human body, amounting to nearly 24 trillion and are produced at a rate of 200 million per day [8]. RBCs’ main function is to deliver oxygen to cells, taking up oxygen in the lungs and releasing it while passing through the body’s capillaries. Moreover, they carry CO_2_ from tissues to lungs. Their cytoplasm is rich in hemoglobin (making up 98% of the non-water content of RBCs’ cytoplasm), an iron-containing molecule that can bind oxygen and gives the characteristic red color to the blood [17]. When completely saturated with oxygen, a RBC can transport up to 1 billion molecules of oxygen [18].

Mammalian erythrocytes are unique among vertebrates as, in their mature form, they are non-nucleated cells and do not possess organelles [11]. Thus, their only structural component is the semi-permeable outer membrane responsible for their shape and deformability. This membrane comprises an outer lipid bilayer, which embeds several proteins that bind a membrane-skeleton network anchored beneath it [19]. Being highly elastic, it quickly responds to applied fluid stresses.

**Figure 1:**
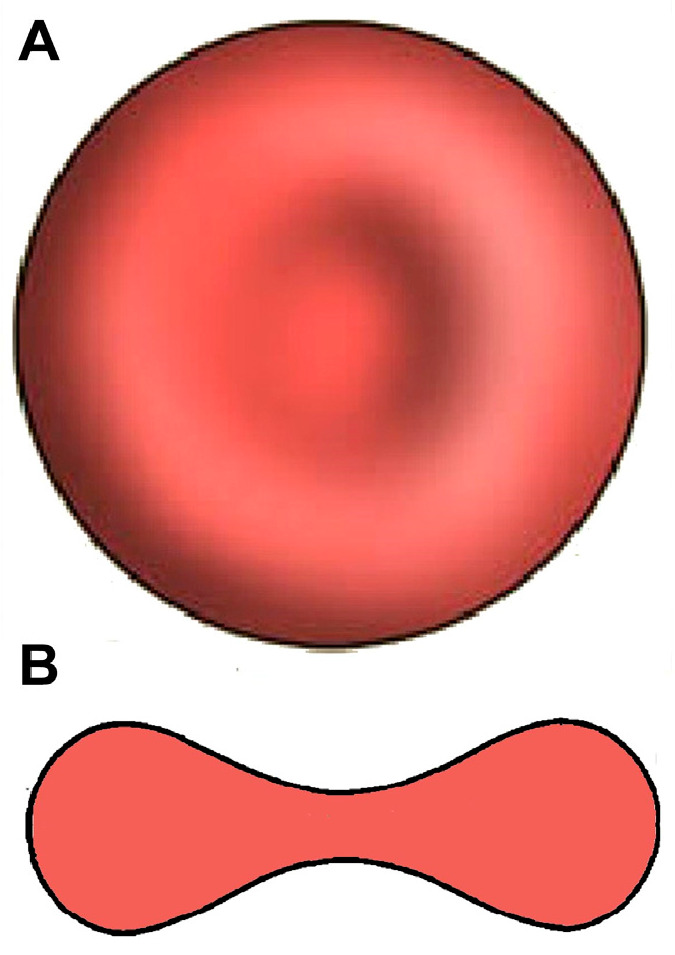
Erythrocyte geometry: (**A**) Top view; (**B**) cross section.

In the vast majority of mammalian species [9], the typical shape is the biconcave disc, flattened and depressed in the center, with a torus-shaped rim on the edge of the disc and with a dumb bell-shaped cross section (Figure 1). Its distinctive biconcave shape results in a high surface-area-to-volume (SA/V) ratio (human RBCs, for example, show a 40% excess surface area compared to a sphere with the same volume). This, together with the weak bending and shear elasticities of the membrane, determines the high deformability of RBCs. Thus, the deformability and mechanical stability of the membrane allows the erythrocyte to undergo extensive passive deformation [20]: for example, mammalian RBCs (with diameters up to 9 μm) can pass through capillaries with a diameter of ~2.5 μm [21] and even through 1.0 μm wide splenic inter-endothelial slits [22,23].

Moreover, erythrocytes’ ability to rapidly change shape in response to fluid shear stress is due to their intracellular viscosity. The latter depends on RBC’s cytoplasmic hemoglobin concentration, and the difference in viscosity between RBC and plasma affects the amount of possible deformation. An increase in hemoglobin concentration allows greater oxygen transport, but above the optimal value it will reduce the cell deformability, since cytoplasmic viscosity increases exponentially as the intracellular hemoglobin concentration rises [24]. Indeed, the cytoplasmic hemoglobin concentration is nearly constant in all mammals, and it has been proposed that this reflects a balance between the advantage of increased oxygen capacity and the disadvantage of increased viscosity [9].

During the different stages of erythroid differentiation, erythroblasts (the erythrocyte precursors) gradually lose volume [25], become anucleated, and finally, during the maturation from reticulocyte to erythrocyte, shrink further and attain a biconcave shape [26]. In the past, it was also believed that RBCs become more spherical with aging because of the selective loss of their extra surface area, while maintaining constant volume [27]. However, it was later clarified that a tightly matched decrease in membrane area and cell volume must exist [28], so that the RBC becomes smaller without losing its biconcave shape and therefore remains deformable. Old RBCs do in fact appear to be less deformable (and thus are more likely to become trapped in the red pulp of the spleen), but that is due to the increased concentration of intracellular Hb, with increased cytoplasmic viscosity [29,30]. Thus, the SA/V ratio is an important parameter that appears to be kept constant during RBC aging in the circulation, in order to unify rheological properties of RBCs of different ages [31]. It should be added that another recent article proposed that the SA/V ratio is the key factor (being more important than intracellular viscosity and elasticity of the membrane) in determining the ability of RBCs to deform and pass through small capillaries [32].

It is intuitive that an increased surface allows a greater exchange of gases. Indeed, it has been shown that the biconcave shape of RBCs allows faster membrane transport and reduces the time delays associated with intracellular gas transport [33]. Moreover, it has been proposed that RBC surface area and hemoglobin-oxygen affinity coevolved in order to satisfy the metabolic demands of mammalian species [9]. Therefore, not only variations in the hemoglobin-oxygen affinity but also in the SA/V ratio can affect the ability of blood to carry and distribute oxygen in the organism.

Reduction of the SA/V ratio results in decreased cellular deformability, compromised red cell function, osmotic fragility and lessened survival. This is the case of hereditary spherocyosis, hemolytic anemias, sickle cell disease, thalassemia, malaria, septicemia, and diabetes mellitus [34]. Indeed, *Plasmodium*-infected RBCs (increasing both volume and surface area) have a decreased SA/V ratio, becoming spherical and rigid [35]. In general, all pathological conditions that increase the sphericity of the RBC (collectively defined as spherocytosis in hematology) lead to a decrease of the SA/V ratio, with a consequent decreased deformability and inability to pass through narrow passages. In this way, RBCs are sequestered and destroyed in the red pulp of the spleen [22], leading to anemia. However, accumulation of RBCs in the capillary beds of the spleen or the brain can even lead to splenic rupture and cerebral injury. Thus, knowledge of the volume and surface area of erythrocytes is essential to evaluate their rheological properties.

The surface area of RBCs is usually measured with the micropipette technique [36,37]. RBCs are placed in a U-shaped chamber, and a micropipette with a diameter of 1.0–1.5 μm is inserted into the chamber through the open side of the “U”. By aspiring RBCs at a pressure of ~6000 dyne/cm^2^, cells are deformed into a sphere plus a cylindrical projection into the micropipette. In this way, measuring the outside diameter and the projection length, the surface area and volume can be calculated. This technique provides a precise measurement of the surface area; however, beside being time-consuming and requiring expertise, it needs instrumentation that is often not available to zoologists, veterinarians or, in general, researchers working in the field. The aim of this paper is to provide a simple model that can be used as the best approximation to determine the erythrocyte surface area by knowing only the diameter and MCV (which, as said before, can be easily measured).

## 2. Materials and Methods

The calculation of the red blood cell volume is a simple method, part of the standard complete blood count. It is called Mean Corpuscular Volume (MCV) in hematology, is expressed in femtoliters (fL = 10^−15^ L = µm^3^) and is calculated using the following formula:

MCV = 10 × hematocrit/RBC count.(1)

The hematocrit is the volume percentage (%) of RBCs in blood, and the RBC count is measured in millions/µL.

**Figure 2:**
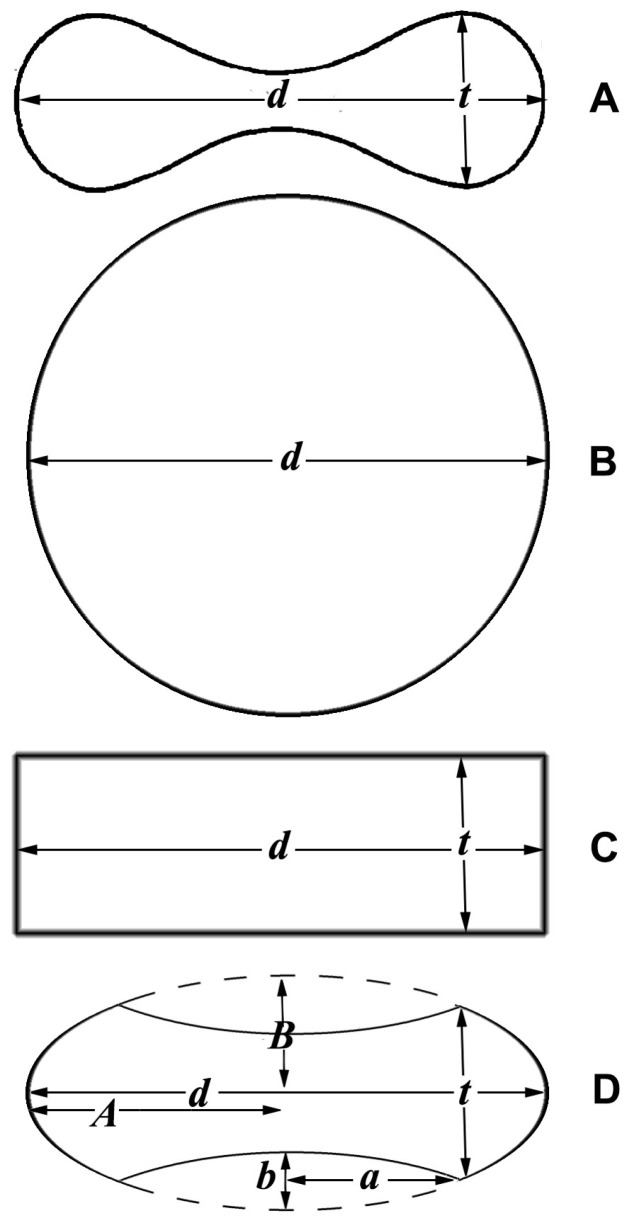
Cross sections of different solids: (**A**) Erythrocyte; (**B**) sphere; (**C**) cylinder; (**D**) spheroid with concave caps; *d* = diameter; *t* = thickness; *A* = major axis of the large spheroid; *B* = minor axis of the large spheroid; *a* = major axis of the small spheroid (cap); *b* = minor axis of the small spheroid.

The most precise description of the erythrocyte geometry is given by the equation of the oval of Cassini, following the physical explanation presented by Canham [38]. The main problem with the formulas proposed so far is the complexity of the mathematical expressions, which comprise elliptic integrals of the first and second kind [39]. For practical purpose, it would be useful to find approximated models that give formulas based on diameter and thickness (Figure 2A). It has been proposed that one consider that the diameter squared of an erythrocyte determines the size of its surface area, while the thickness does not have a major influence [40]. This means considering the erythrocytes as a sphere (Figure 2B). Beside this model, I also propose a cylinder (Figure 2C) and an oblate spheroid with concave caps (Figure 2D).

For the sphere, the formulas for volume (V) and surface area (SA) are:

(2) $$V_{s}=\frac{\pi }{6}d^{3},$$

(3) $${SA}_{s}={\pi d}^{2},$$

where *d* is the diameter and *t* the thickness. For the cylinder, the formulas are:

(4) $$V_{c}=\frac{\pi }{4}d^{2}t,$$

(5) $${SA}_{c}=\pi d\left(\frac{d}{2}+t\right).$$

For the spheroid with concave caps, this can be considered as a large ellipsoid of rotation around the minor axis, from which two little ellipsoids, each equal to twice the volume of the concavity, are subtracted [41]. This is described by the following formulas:

(6) $$V_{b}=\frac{4}{3}\pi A^{2}B-2\left(\frac{4}{3}\pi a^{2}b\right),$$

(7) $${SA}_{b}=2\pi A^{2}+2\pi AB\frac{\sinh^{-1}e}{e},$$

where *e* is the eccentricity around the minor axis, equal to $\frac{\sqrt{A^{2}-B^{2}}}{B}$, *A* is the major semi-axis of the large spheroid and corresponds to half the diameter of the cell (*d*), *B* is the minor semi-axis of the large spheroid, and *a* and *b* are the major and minor semi-axes, respectively, of the small spheroids (Figure 2D). Approximating *B* as 2/3 of the thickness (*t*), *b* as 1/8 of *t* and *a* as 1/4 of the diameter (*d*) [41], the formulas (6) and (7) can be written as follows:

(8) $$V_{b}=\frac{2}{3}\pi t\left(\frac{d^{2}}{3}-\frac{t^{2}}{32}\right),$$

(9) $${SA}_{b}=\pi d\left(\frac{d}{2}+\frac{2}{3}t\frac{\sinh^{-1}e}{e}\right).$$

## 3. Results and Discussion

**Figure 3:**
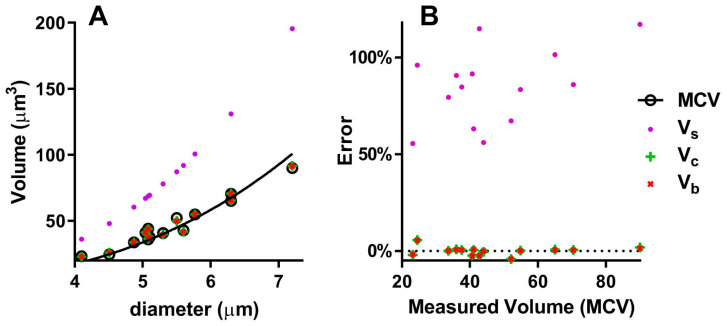
Comparison of different models to calculate RBC volume: (**A**) Correlations of measured and calculated volumes with diameter; (**B**) percentage errors of calculated volumes; MCV = Mean Corpuscular Volume (measured), V_s_ = Volume of the sphere, V_c_ = Volume of the cylinder, V_b_ = Volume of the biconcave spheroid.

To test the different models approximating the geometry of the RBC (sphere, cylinder, spheroid with concave caps), we can use the few works where the diameter, thickness and volume are directly measured. Table 1 compares the volumes obtained with the three models to the volumes measured as MCV in different mammalian species. The volume of the sphere is highly divergent from the measured volume, with a Mean Absolute Percentage Error (MAPE) of 84.8% (Table 1 and Figure 3B). In fact, the correlation curves of V_s_ and MCV (Figure 3A) are significantly different (*p* < 0.0001). Instead, the volumes of the cylinder and the spheroid with concave caps are nearly identical, with a 1.6% and 1.5% MAPE (Table 1) and give a good approximation of the real volume, both with small and large erythrocytes (Figure 3): the differences between V_c_ and MCV and between V_b_ and MCV are not statistically significant (*p* = 0.953 and *p* = 0.982, respectively).

**Figure 4:**
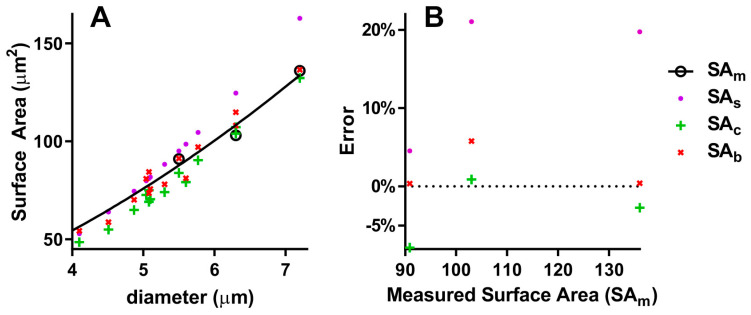
Comparison of different models to calculate RBC surface area: (**A**) Correlations of measured and calculated surface areas with diameter; (**B**) percentage errors of calculated surface areas; SA_m_ = measured surface area, SA_s_ = Surface area of the sphere, SA_c_ = Surface area of the cylinder, SA_b_ = Surface area of the biconcave spheroid.

Unfortunately, the number of species used for direct measures of surface area (SA_m_) is very small (Table 2). Nonetheless, I present a comparison of these with the calculations obtained by the above formulas. As can be seen, the sphere model always gives an overestimation, with a 15.1% MAPE (Table 2 and Figure 4B), and the correlation curves of SA_s_ and SA_m_ (Figure 4A) are significantly different (*p* < 0.0001). The cylinder model gives a 3.2% MAPE, and the correlation curves of SA_c_ and SA_m_ are significantly different (*p* = 0.0178). Finally, the biconcave spheroid gives a 1.9% MAPE, and the correlation curves of SA_b_ and SA_m_ are not significantly different (*p* = 0.8805).

From these data, it seems that thickness is a relevant measure in the determination not only of the volume but also of the surface area. Therefore, the spherical model should be rejected. While the volumes obtained with the cylinder and the biconcave spheroid models give a good approximation of the real volume and are nearly identical to each other, the latter model seems more accurate in order to calculate the surface area.

It can be concluded that the cylinder model (which is easier to adopt) can be used for calculating the RBC volume, while the biconcave spheroid shall be used for calculating the surface area.

**Figure 5:**
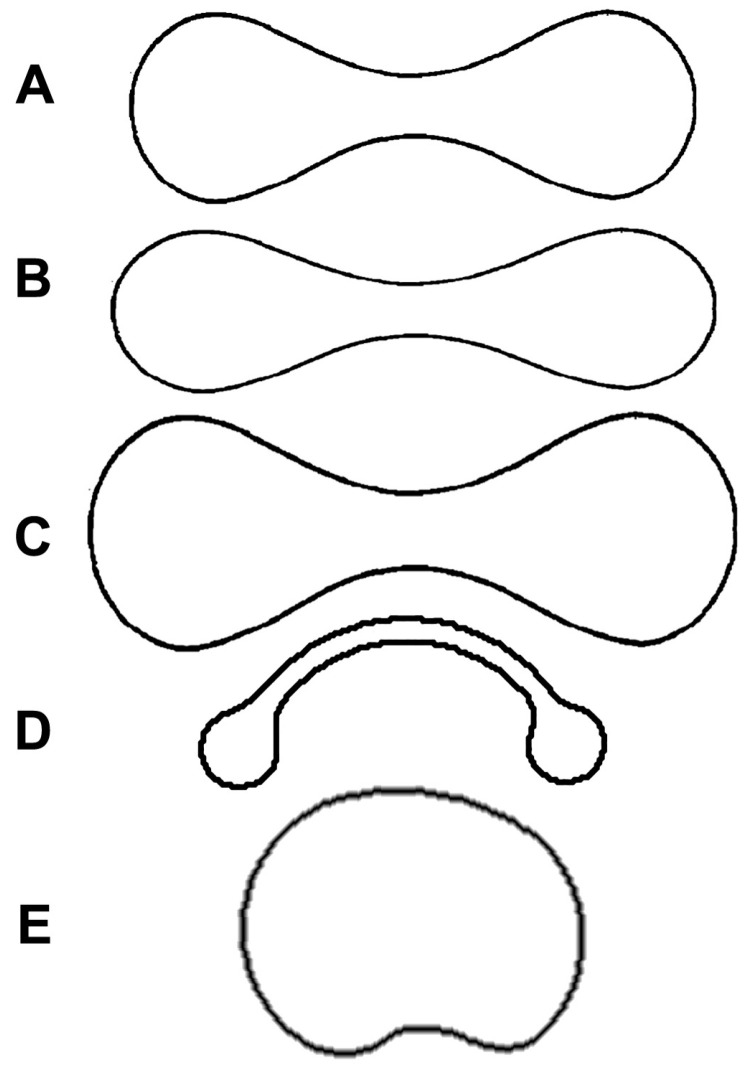
Cross sections of normal and abnormal RBCs: (**A**) Normal erythrocyte (discocyte); (**B**) leptocyte (increased diameter, but normal volume); (**C**) macrocyte (increased diameter and volume); (**D**) codocyte (bell-shaped); (**E**) spherocyte.

However, this protocol assumes that RBCs have the normal biconcave shape (being known in hematology as discocytes) and may be inadequate when pathological or species-specific features give place to different shapes. Therefore, I tested the protocol with human abnormal RBCs (Figure 5) and with musk deer (*Tragulus javanicus*) ones. Starting from data of MCV and diameter, I calculated the thickness and surface area (Table 3). As can be seen, when RBCs are spherical, as in the case of patients with Hereditary Spherocytosis and *Tragulus javanicus* [54], the thickness: diameter ratio is decisively higher than in the other cases. In one case, the surface area is not even calculable (Table 3) because the eccentricity becomes an imaginary number. Therefore, I suggest two rules of thumb. First, when measuring RBC diameter on blood smear, check for the central pallor in the cells (caused by the RBC being thinner in the biconcavity): if it is absent, RBCs are probably spherical, and the model should be rejected. Second, if the calculated thickness: diameter ratio is 0.5 or greater, the model is also invalid in this case. If the model is rejected because RBCs are spherical, their surface area can be easily calculated using the formula (2) for the sphere. Therefore, the correct value of the surface area for *Tragulus javanicus* is 15.2 (not 24.7) µm^3^.

Therefore, among mammalian species, two important exceptions must be considered. First, the musk deer *Tragulus javanicus* shows the smallest erythrocytes, which are actually spherical [54]: as said above, for this species, the formulas of the spherical model should be used. Second, camelids (camels, llamas and alpacas) show characteristic elliptic erythrocytes that are broad and flattened, without concavity [16]. For these animals, I propose to use the formulas of the elliptical cylinder:

(10) $$V=\frac{\pi }{4}abt,$$

(11) $$SA=\frac{\pi }{2}\left(a+b\right)t+\frac{\pi }{2}ab,$$

where *a* and *b* are the major and minor diameters.

## 4. Conclusions

As stated earlier, volume (MCV) and diameter are easily obtained from a complete blood count and observations of blood smears, respectively. Thickness and surface area, instead, are much more difficult to measure. Therefore, the following steps are proposed:

1. Determination of the MCV (i.e., the RBC volume): this can be done with an automatic hematology analyzer (or, alternatively, by measuring the hematocrit (Hct) with a micro-hematocrit tube and a centrifuge) and by determining the cell count (RBC) in the same sample. The MCV can be obtained by dividing the Hct by the cell count: MCV (fl) = Hct/RBC. Example: Hct = 0.45; RBC = 5.5 × 10^6^ RBC/µL. MCV = (0.45/5.5 × 10^6^ RBC/µL) × (10^9^ fl/µL) = 81.8 fl;
2. Measure of the diameter (*d*): as hematology analyzers do not give this value, it should be obtained manually by measuring at least 100 cells in a blood smear. In order to avoid possible artefacts associated with the smearing procedure itself, only round cells with central pallor (thus meaning that they are orientated parallel to the microscope slide plane) should be scored;
3. Calculation of the thickness: $t=\frac{4MCV}{\pi d^{2}}$ (Equation (4)) (concerning the volume, the cylinder model is nearly identical to the spheroid, Equations (6) and (8), but is far simpler);
4. Calculation of the Surface Area: $SA=\pi d\left(\frac{d}{2}+\frac{2}{3}t\frac{\sinh^{-1}e}{e}\right)$ (Equation (9)).

In this way, all the main geometric quantities of the erythrocyte are obtained, and they can be used to assess variations of SA, SA/V ratio, etc., in many fields, such as comparative hematology, biorheology and veterinary medicine.

## Figures and Tables

**Table 1:** Observed and calculated erythrocyte volumes in different species.

Species	*d*	*t*	MCV	*V_s_*	*V_c_*	*V_b_*	Reference
*Capra hircus* (goat)	4.1	1.7	23.2	36.1	22.7	22.8	[16]
*Sorex Araneus* (shrew)	4.5	1.6	24.5	48	25.9	25.9	[42]
*Microtus subterraneus* (pine vole)	4.9	1.8	33.7	60.5	33.7	33.7	[43]
*Lagurus lagurus* (steppe lemming)	5.1	1.8	36	68.6	36.3	36.2	[43]
*Myodes glareolus* (bank vole)	5.1	1.9	37.6	69.5	37.8	37.8	[44]
*Otomops martiensseni* (free-tailed bat)	5.3	1.8	40.7	77.9	39.7	39.6	[45]
*Microtus agrestis* (field vole)	5.0	2.1	41.1	67	41.3	41.4	[43]
*Sicista betulina* (birch mouse)	5.6	1.7	42.8	91.9	41.9	41.7	[46]
*Microtus oeconomus* (tundra vole)	5.1	2.2	44	68.6	43.8	43.9	[47]
*Mus musculus* (house mouse)	5.5	2.1	52.1	87.1	49.9	49.9	[48]
*Bison bonasus* (European bison)	5.77	2.1	54.8	100.6	54.9	54.9	[49]
*Rattus norvegicus* (brown rat)	6.3	2.1	65	130.9	65.5	65.3	[50]
*Oryctolagus cuniculus* (rabbit)	6.3	2.3	70.4	130.9	70.8	70.7	[51]
*Homo sapiens* (human)	7.2	2.2	90	195.4	91.6	91.3	[52]
**MAPE**				**84.8%**	**1.6%**	**1.5%**	

*d* = mean diameter, *t* = mean thickness, MCV = Mean Corpuscular Volume, *V_s_* = Volume of the sphere, *V_c_* = Volume of the cylinder, *V_b_* = Volume of the biconcave spheroid, MAPE = Mean Absolute Percentage Error.

**Table 2:** Observed and calculated erythrocyte volumes in different species.

Species	*SA_m_*	*SA_s_*	*SA_c_*	*SA_b_*	Reference
*Capra hircus* (goat)	n.a.	52.8	48.6	54.4	[16]
*Sorex Araneus* (shrew)	n.a.	63.9	54.9	58.7	[42]
*Microtus subterraneus* (pine vole)	n.a.	74.5	64.9	70.2	[43]
*Lagurus lagurus* (steppe lemming)	n.a.	81.1	69.1	73.5	[43]
*Myodes glareolus* (bank vole)	n.a.	81.7	70.5	75.6	[44]
*Otomops martiensseni* (free-tailed bat)	n.a.	88.3	74.1	78.1	[45]
*Microtus agrestis* (field vole)	n.a.	79.8	72.7	80.9	[43]
*Sicista betulina* (birch mouse)	n.a.	98.5	79.2	81.1	[46]
*Microtus oeconomus* (tundra vole)	n.a.	81.1	75	84.4	[47]
*Mus musculus* (house mouse)	90.9	95	83.8	91.2	[36]
*Bison bonasus* (European bison)	n.a.	104.6	90.4	97	[49]
*Rattus norvegicus* (brown rat)	103	124.7	103.9	108.1	[53]
*Oryctolagus cuniculus* (rabbit)	n.a.	124.7	107.8	114.9	[51]
*Homo sapiens* (human)	136	162.9	132.3	136.5	[52]
**MAPE**		**15.1%**	**3.2%**	**1.9%**	

*SA_m_* = measured surface area, *SA_s_* = Surface area of the sphere, *SA_c_* = Surface area of the cylinder, *SA_b_* = Surface area of the biconcave spheroid, n.a. = not available, MAPE = Mean Absolute Percentage Error.

**Table 3:** Application of the proposed method in different types of RBCs.

Species/Type	Measured MCV	Measured RBC Diameter	Calculated RBC Thickness	Calculated RBC Surface Area	Thickness: Diameter Ratio	Is Biconcave Model Valid?
*Homo sapiens*						
Discocyte [52]	90	7.2	2.2	136	0.3	yes
Macrocyte [55]	120	9.0	1.9	176.8	0.2	yes
Leptocyte [55]	90	8.5	1.6	150.2	0.2	yes
Codocyte [55]	60	6.0	2.1	106.4	0.4	yes
Spherocyte [55]	90	4.0	7.2	n.c.	1.8	no
HS patients [56]	80	5.9	3.0	136.1	0.5	no
*Tragulus javanicus* [54]	5.6	2.2	1.5	24.7	0.7	no

HS: Hereditary spherocytosis; n.c.: not computable. Superscripts show references for measured MCV and diameter.

## Data Availability

No additional data were created or analyzed in this study. Data sharing is not applicable to this article.
